# Upcycling Black Garlic Peels into Multifunctional Cosmeceutical Extracts: Antioxidants and UV-Shielding via Antimicrobial Natural Deep Eutectic Solvents

**DOI:** 10.3390/antiox15060671

**Published:** 2026-05-27

**Authors:** Filippo Marchetti, Ilenia Gugel, Irene Gugel, Valentina Vecchi, Giuseppe Sabbioni, Anna Baldisserotto, Stefania Costa, Monica Borgatti, Stefano Manfredini, Silvia Vertuani

**Affiliations:** 1 Department of Life Sciences and Biotechnology, University of Ferrara, Via Fossato di Mortara 17–19, 44121 Ferrara, Italy; ilenia.gugel@unife.it (I.G.); valentina.vecchi@unife.it (V.V.); giuseppe.sabbioni@unife.it (G.S.); anna.baldisserotto@unife.it (A.B.); stefania.costa@unife.it (S.C.); monica.borgatti@unife.it (M.B.); smanfred@unife.it (S.M.); silvia.vertuani@unife.it (S.V.); 2 Interuniversity Consortium for Biotechnology (CIB), 34100 Trieste, Italy

**Keywords:** black garlic peels, natural deep eutectic solvents, sunscreen, antioxidants, multifunctional extracts

## Abstract

Black garlic peels (BGPs) are a largely underutilized by-product despite representing an untapped source of bioactive compounds. This study presents a sustainable upcycling protocol for black garlic peels, evaluating natural deep eutectic solvents (NaDES) to develop multifunctional extracts for green cosmetics. Following the screening of four eutectic mixtures, the choline chloride–lactic acid (ChCl:LA) system demonstrated the highest extraction efficiency. The optimized extract yielded a remarkable total phenolic content (5216.61 µg GAE/mL) and strong antioxidant capacity, confirmed by DPPH and FRAP assays, associated with recovering both free and bound phenolic fractions. Subsequent HPLC profiling characterized the extract, and the comparative analysis explicitly demonstrated that antimicrobial activity is entirely driven by and identical to the pure eutectic solvent vehicle rather than the extracted garlic biomass, with broad-spectrum efficacy against *C. albicans*, *P. aeruginosa*, and *S. aureus*. To evaluate its cosmeceutical potential, the extract was incorporated into emulsions (5%, 10%, 15% *w*/*w*) with inorganic or organic UV filters. Although the direct Sun Protection Factor (SPF) and the UVA Protection Factor (UVA-PF) did not show enhancing results, a photochemiluminescence (PCL) analysis revealed a synergistic behaviour with organic filters, successfully boosting the formulation’s biological antioxidant shield. This pioneering work highlights BGP’s upcycling potential, proposing NaDES extracts as highly promising multifunctional, antioxidant, and antimicrobial ingredients for next-generation cosmeceuticals.

## 1. Introduction

*Allium sativum* L. is globally recognized as a plant species with extensive applications in both human nutrition and health. Black garlic (BG) is derived from fresh garlic (FG) through controlled thermal processing under high temperature and humidity, and this transformation induces significant qualitative and quantitative modifications in the phytochemical composition of FG [1,2,3], shifting its chemical composition toward a higher concentration of bioactive compounds with antioxidant, anti-inflammatory, hypocholesterolaemic, anti-atherosclerotic, hypoglycaemic, and antimicrobial properties [2,4,5]. Although the health-promoting properties, food applications, and biochemical profile of black garlic are extensively documented [6], the valorisation of its processing by-products remains underexplored, particularly regarding the inner and outer peels. The industrial processing of FG generates a substantial volume of waste, with approximately 240 g of inner and outer peels produced for every kilogram of fresh bulbs processed. Garlic peels account for nearly 25% of the raw material’s total mass. Given that this translates to an estimated global accumulation of 2.3 to 2.9 million tons of discarded agricultural biomass annually, the development of sustainable and high-value valorisation strategies for this agro-industrial by-product has become a critical priority [7]. While fresh garlic residues have been successfully repurposed in the context of lithium-sulphur batteries, in the synthesis of porous carbon for supercapacitors, for the biosorption of tungstate, and in the development of functional packaging [8,9,10,11,12], BG peels represent a largely neglected biomass. Despite this lack of characterization, BG peels hold substantial potential as a sustainable source of high-value bioactive compounds with potent antioxidant and therapeutic activities.

It is important to emphasize that within the framework of biomass valorisation for the recovery of valuable bioactives, the selection of an appropriate extraction methodology, aligned with *green chemistry* metrics, has become imperative to design systems that are both highly efficient and sustainable. Recently, the processing of biomass using modern, environmentally friendly technologies has increasingly focused on natural deep eutectic solvents (NaDESs). These tunable mixtures typically consist of two or three naturally occurring components functioning as hydrogen bond acceptors (HBAs) and hydrogen bond donors (HBDs) [13]. NaDESs offer profound advantages: they are safe, highly efficient, often exhibiting performance comparable to conventional hydroalcoholic solvents [14], and biocompatible [15]. NaDESs have emerged as inherently biodegradable and non-volatile green solvents, exhibiting high efficiency in the solubilization of a broad spectrum of bioactive molecules, including polyphenols, flavonoids, anthocyanins, carotenoids, and alkaloids [16]. Notably, their very low to negligible toxicity often allows for direct incorporation into final formulations, thereby bypassing resource-intensive isolation and purification steps [17]. Furthermore, coupling NaDESs with ultrasound-assisted extraction (UAE) yields synergistic benefits. Specifically, NaDES-mediated UAE mitigates the critical bottlenecks of conventional extractions by lowering operational temperatures and drastically reducing extraction times. When applied to the recovery of valuable compounds, this integrated framework represents an effective model of upcycling, successfully translating circular economy principles into the generation of entirely sustainable biologically active extracts [18,19,20,21].

Considering these premises, this study proposes, for the first time, a targeted upcycling protocol for black garlic peels employing NaDES-mediated ultrasonic extraction. This approach successfully yielded extracts with a pronounced antioxidant capacity; notably, the application of a choline chloride–lactic acid mixture maximized the overall antioxidant performance. Furthermore, a broad-spectrum antimicrobial activity was identified against *S. aureus*, *P. aeruginosa*, and *C. albicans*; comparative evaluations explicitly revealed that the native ChCl:LA eutectic mixture alone drives this biocidal efficacy at the tested concentrations, rather than the BGP extracts. Finally, the potential of ChCl:LA BGP extracts was tested as photoprotective boosters when incorporated into formulations alongside organic and inorganic UV filters. Although no direct or synergistic photoprotective activity was observed for the extracts, neither alone nor in combination with the sunscreens, the ChCl:LA BGP extracts demonstrated the ability to boost the antioxidant efficacy when combined with the organic filters evaluated in this study. To the best of our knowledge, this innovative research represents a model for sustainable and circular approaches in modern cosmetic and cosmeceutical formulations.

## 2. Materials and Methods

### 2.1. Materials

Black garlic peels were manually separated from cloves provided by a local producer (NeroFermento ^®^, Ravenna, Italy). All reagents were purchased from Merck (Milan, Italy), and all the solvents used in this research were purchased from Carlo Erba (Milan, Italy).

### 2.2. Natural Deep Eutectic Solvent (NaDES) Preparation

NaDESs were prepared using the heating-and-stirring method. Briefly, individual components were accurately weighed and combined in specific molar ratios. Deionized water was added to the mixture at defined concentrations (*w*/*v*%). The mixtures were then heated at 50 °C and stirred at 500 rpm until a homogeneous, clear, viscous liquid was formed [22]. Depending on the starting components (Table 1), the resulting solvents exhibited a characteristic yellowish to brownish appearance. The formation of each NaDESs at varying water concentrations was qualitatively validated via Fourier-transform infrared spectroscopy (FT-IR) using a PerkinElmer Spectrum spectrophotometer (PerkinElmer, Waltham, MA, USA) equipped with an attenuated total reflectance (ATR) accessory. The resulting spectra of the eutectic mixtures were compared against those of the individual starting components to confirm the establishment of the hydrogen-bonded network and the structural integrity of the solvent, as reported in our previous study [23].

### 2.3. General Extraction Procedure

The extraction of bioactive compounds from black garlic peels was performed using an ultrasound-assisted extraction procedure following a modified protocol reported in Bravi et al. [24]. Briefly, 0.200 g of BGP was placed in a capped centrifuge tube and homogenized with the extraction mixture at a solid-to-solvent ratio of 1:10 *w/v*. The ultrasonic bath (Ceia CP104, CEIA, Viciomaggio, Italy) was operated at a frequency of 39 kHz and a temperature of 60 °C for 40 min. Following the extraction, the mixture was centrifuged to separate the supernatant from the solid residue. The resulting extract was stored at 4 °C in the dark until further analysis.

The remaining solid fraction underwent alkaline hydrolysis [24] to release bound phenolic compounds. The residue was incubated with 4 M NaOH at a 1:10 ratio and subjected to a second UAE cycle under the aforementioned conditions (39 kHz, 60 °C, 40 min). The hydrolysis was allowed to proceed overnight at room temperature. Subsequently, the mixture was acidified with 4 M HCl to pH 2, and the hydrolysed phase was then extracted three times with ethyl acetate (1:3 *v*/*v* per cycle). The combined organic layers were dried over anhydrous sodium sulphate, filtered, and evaporated to dryness under reduced pressure at 40 °C. The dry residue was finally reconstituted in methanol and stored at 4 °C in the dark prior to subsequent determinations. Conventional solvent extractions using aqueous ethanol and methanol (80% *v*/*v*) were performed under identical conditions to serve as benchmarks for comparison with the NaDES performance. Three separate extraction procedures for every experimental set were performed under identical conditions starting from the same batch of dried BGP material.

### 2.4. Determination of Total Polyphenols

The total polyphenol content (TPC) of each extract was determined using the Folin–Ciocalteu method, following the protocol described by Singleton et al. [25], with minor modifications. A calibration curve was constructed using gallic acid as the standard within a concentration range of 1–20 µg/mL. Briefly, each extract or standard solution was treated with a diluted aqueous Folin–Ciocalteu reagent. The mixture was vortexed for 10 s and subsequently incubated in the dark for 5 min. Following this, 300 µL of a 20% *w*/*v* Na_2_CO_3_ solution was added to each tube. The samples were then incubated for 90 min in the absence of light to allow for colour development. The absorbance was measured at 765 nm against a blank containing distilled water. TPC results were expressed as micrograms of gallic acid equivalents per mL of extract (µg GAE/mL). All measurements were performed in triplicate from independent extraction replicates (n = 9), and the results were reported as the mean value ± standard deviation (SD), as previously described in [26].

### 2.5. DPPH Antioxidant Potential

The antioxidant capacity was evaluated via the 2,2-diphenyl-1-picrylhydrazyl (DPPH) radical scavenging assay according to Wang et al. [27]. The method relies on the decolorization of the DPPH radical from purple to pale yellow upon reduction by antioxidant compounds.

Briefly, 1.5 mL of extract was mixed with 0.750 mL of a DPPH methanolic solution. The mixture was vortexed and incubated at room temperature in the dark for 30 min. The absorbance was recorded at 517 nm. The radical scavenging activity was calculated using the following equation:

$$IC\;\%=\left\lfloor 1-\frac{\left(A_{1-}A_{2}\right)}{A_{0}}\right\rfloor \times 100$$

where A_0_ represents the absorbance value of the control (DPPH solution without sample), A_1_ is the absorbance of the sample reacted with DPPH, and A_2_ is the absorbance of the sample blank (sample without DPPH) to account for inherent matrix interference.

### 2.6. Ferric Reducing Antioxidant Power (FRAP) Assay

The reducing capacity of the extracts was determined using the FRAP assay, according to [28]. The assay measures the reduction of the Fe(III)-TPTZ complex to the blue-coloured Fe(II)-TPTZ complex in an acidic environment. The FRAP reagent was freshly prepared by mixing 0.1 M acetate buffer (pH 3.6), 10 mmol/L TPTZ in 40 mmol/L HCl, and 20 mmol/L ferric chloride in a 10:1:1 ratio. For the analysis, 100 µL of sample (or solvent for the blank) was added to 1.9 mL of the FRAP reagent. The mixture was vortexed and incubated at 37 °C in the dark for 10 min. The absorbance was measured at lambda = 593 nm using a UV–vis spectrophotometer (Beckman Coulter s.r.l., Milano, Italy). Quantification was performed using a Trolox calibration curve, and results were expressed as micromoles of Trolox equivalents per gram of extract (µmol TE/g). Each assay was performed at least in triplicate from independent extraction replicates (n = 9), and the results were reported as the mean value ± SD.

### 2.7. HPLC Determination of Phenolic Compounds

The polyphenolic profile of the extracts was characterized following the methodology described by Baldisserotto et al. [26]. Chromatographic analyses were conducted using an Agilent 1100 Series High-Performance Liquid Chromatography system (Agilent Technologies, Santa Clara, CA, USA), equipped with a G1315A Diode Array Detector (DAD) configured to monitor absorbance at 254 ± 8 nm. Analyte separation was achieved at ambient temperature on a Synergi Hydro-RP C18 column (250 mm × 4.6 mm, i.d., 4 µm particle size, 80 Å pore size; Phenomenex, Torrance, CA, USA). The mobile phase consisted of 0.1% *v*/*v* trifluoroacetic acid (TFA) in ultrapure water (Solvent A) and 0.1% *v*/*v* TFA in acetonitrile (Solvent B), delivered at a constant flow rate of 1.2 mL/min. A multi-step gradient elution program was applied, initiating with a linear gradient from 90% to 80% A over 5 min, followed by an isocratic hold at 80% A for 5 min. The gradient was then linearly decreased from 80% to 20% A over 10 min and finally returned rapidly from 20% to 90% A in 2 min to allow for column re-equilibration. Prior to injection, all samples and standard solutions were clarified by filtration through 0.22 µm cellulose acetate syringe filters. A standardized injection volume of 5 µL was utilized for all analytical runs. For quantitative evaluation, a composite stock solution of reference standards was prepared in a 1:1 (*v*/*v*) water-to-acetonitrile mixture. This standard mix comprised ferulic acid (1.06 mg/mL), gallic acid (1.58 mg/mL), chlorogenic acid (0.23 mg/mL), rutin (0.325 mg/mL), caffeic acid (0.120 mg/mL), quercetin (0.50 mg/mL), ellagic acid (0.086 mg/mL), gentisic acid (0.99 mg/mL), and salicylic acid (1.11 mg/mL). The stock standard solution was appropriately diluted to construct calibration curves for the targeted quantification of each compound.

### 2.8. Antimicrobial Activity

The antimicrobial activity of the serially diluted BGP ChCl:LA extract was evaluated via the broth macrodilution method. *Candida albicans* DSM 1386, *Staphylococcus aureus* DSM 799, and *Pseudomonas aeruginosa* DSM 1128 (Leibniz Institute DSMZ-German Collection of Microorganisms and Cell Cultures GmbH, Braunschweig, Germany) were used. For antifungal assay, *C. albicans* was cultured overnight in yeast extract broth at 28 °C under continuous agitation (100 rpm). The inoculum was then standardized using RPMI 1640 broth to achieve a final concentration ranging from 5.0 × 10^2^ to 2.5 × 10^3^ cells/mL. MIC determination was performed in accordance with the CLSI M27-A3 [29] guidelines, with tubes incubated for 24 h at 35 °C. For the antibacterial evaluation, *S. aureus* and *P. aeruginosa* were grown overnight in tryptone soya yeast extract medium at 37 °C and 100 rpm. The bacterial inocula were adjusted to a final working concentration of 1 × 10^6^ CFU/mL. The MIC assay was conducted following the CLSI M07-A9 [30] guidelines, with an incubation period of 24 h at 37 °C. In all assays, the MIC was defined as the lowest concentration of the extract that completely inhibited visible microbial growth (absence of turbidity). Visual assessments were analytically confirmed by measuring the spectrophotometric absorbance of the suspensions at 530 nm for *C. albicans*, and at 600 nm for both *S. aureus* and *P. aeruginosa*. MIC determination was performed in 3 independent assay replicates for each microorganism, each confirmed by triplicate spectrophotometric readings. The results are reported as mean value ± SD.

### 2.9. Formulation of Sunscreen Emulsions

To evaluate whether the incorporation of the BGP choline chloride–lactic acid eutectic extract (40% water content) could influence photoprotective activity, oil-in-water (O/W) emulsions were developed utilizing the ingredients detailed in Table 2. Specifically, individual emulsions were formulated containing the BGP eutectic extract alone at concentrations of 5%, 10%, and 15% *w*/*w*. To assess potential combinatorial effects, further formulations were prepared combining the eutectic extract (at 5%, 10%, and 15% *w*/*w*) with either a physical UV filter, namely 10% *w*/*w* titanium dioxide (rutile TiO_2_, <100 nm), or a designated organic filter blend comprising 3% butyl methoxydibenzoylmethane and 3% ethylhexyl salicylate.

The formulation process was performed with the separate solubilization of the hydrophilic components (Phase A), the lipophilic components (Phase B), under continuous magnetic stirring, with concurrent heating to a target temperature of 60 °C. Upon reaching thermal equilibrium, the two phases were emulsified utilizing a Polytron™ PT1200E Handheld Homogenizer (Kinematica, Malters, Switzerland). The resulting O/W emulsion was subsequently allowed to cool gradually to room temperature. The filter(s), preservative, and pH modifier (Phase C) were added, and the pH was adjusted to a final value of 5.5 using 10% *w*/*v* solutions of citric acid and NaOH. Control samples were prepared following an identical formulation protocol, yielding a base emulsion devoid of any extracts or UV filters, alongside reference emulsions containing exclusively either inorganic or the organic filters.

### 2.10. Determination of In Vitro Sun Protection Factor

The in vitro SPF of the formulations was evaluated following a previously validated protocol [31], which adapts the ISO 24443:2012 [32] standard for in vitro UVA protection to encompass UVB evaluation, in strict compliance with the European Recommendation EC 647/2006 regarding sunscreen efficacy.

Spectrophotometric measurements of UV absorbance, derived from transmittance data, were performed using a UV-2600 spectrophotometer equipped with an ISR-2600 60 mm integrating sphere (Shimadzu, Milan, Italy). Data acquisition and processing were managed via SPF Calculator software (version 2.1, Shimadzu). The emission spectra were recorded over a wavelength range of 290 to 400 nm, utilizing a scanning step of 1 nm. Sample irradiation was carried out using a SUNTEST CPS+ solar simulator (Atlas, Linsengericht, Germany). The device was equipped with a xenon lamp, a specific optical filter to exclude wavelengths below 290 nm, and an infrared (IR)-blocking filter to prevent unwanted thermal effects. Following the ISO 24443:2012 guidelines, the irradiation intensity was configured to operate within the range of 40 to 200 W/m^2^. SPF and UVA-PF were calculated according to the method of Cesa et al. [33] using the reported Equations (1) and (2):

(1) $$in\;vitro\;SPF=\frac{\int_{\lambda \;=\;290\;nm\;\;\;\;}^{\lambda \;=\;400\;nm}(E\left(\lambda \right)I\left(\lambda \right)d\left(\lambda \right))}{\int_{\lambda \;=\;290\;nm\;\;}^{\lambda \;=\;400\;nm\;}(E\left(\lambda \right)I\left(\lambda \right)10^{-A\left(\lambda \right)}d\left(\lambda \right))}$$

where E(λ) is the erythema action spectrum (CIE-1987) at a wavelength λ, I(λ) is the spectral irradiance received from the UV source at λ, A(λ) denotes monochromatic absorbance per plate before UV exposure of the test product layer at λ, and d(λ) denotes the λ step (1 nm);

(2) $$UVAPF=\frac{\int_{\lambda =290\;nm\;\;\;\;}^{\lambda =400\;nm}(P\left(\lambda \right)I\left(\lambda \right)d\left(\lambda \right))}{\int_{\lambda =290\;nm\;\;}^{\lambda =400\;nm\;}(P\left(\lambda \right)I\left(\lambda \right)10^{-A\left(\lambda \right)C}d\left(\lambda \right))}$$

where I(λ), A(λ), d(λ) are defined by Equation (1), P(λ) is the PPD (Persistent Pigment Darkening) action spectrum, and C is the coefficient of adjustment of the calculated in vitro SPF value to the labelled (in vivo) SPF value (recommended between 0.8 and 1.2). The UVA protection factor (UVAPF) of each sunscreen formulation was instrumentally verified before and after a period of controlled UV irradiation.

### 2.11. Photochemiluminescence Assay

The antioxidant capacity of the samples against superoxide anion radicals was evaluated using the photochemiluminescence (PCL) assay, adapting the methodology described by Popov and Lewin and adapted from a previously reported assay performed in Baldisserotto et al. [26,34]. Briefly, superoxide radicals were optically generated via the photosensitizing agent luminol upon exposure to UV irradiation (Double Bore^®^ phosphorous lamp (Double Bore, Ivrine, CA, USA); emission wavelength 351 nm, irradiance 3 mW/cm^2^). The lipid-soluble antioxidant capacity was quantified utilizing a commercial ACL (Antioxidant Capacity of Liposoluble substances) kit. The kinetic profile of the luminol light emission was continuously monitored over a 180 s interval. Data acquisition and the calculation of the area under the curve (AUC) were performed using the proprietary PCL soft control and analysis software (Version 5.1). The final results were expressed as micromoles of Trolox equivalents per gram of dry sample (µmol TE/g). Prior to analysis, the ultrasound-assisted BGP extracts prepared in ChCl:LA (40% *w*/*v* water content) were suitably diluted to fit within the standard calibration range. Emulsion formulations were subjected to a preliminary extraction phase to isolate the active components. Each assay was performed at least in triplicate from independent extraction replicates (n = 9), and the results were reported as the mean value ± standard deviation (SD).

## 3. Results and Discussions

### 3.1. Free and Bound Polyphenol Content

**Figure 1:**
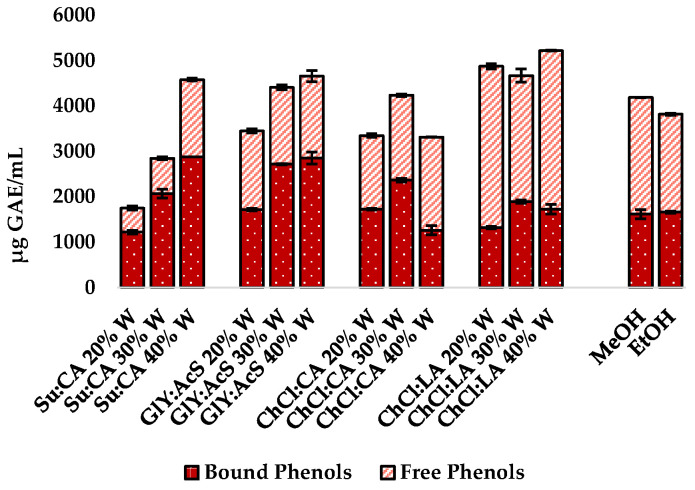
TPC in BGP for different eutectic mixtures evaluated at different water contents (%W).

The efficiency of NaDESs applied to ultrasound-assisted extraction of polyphenols was evaluated and compared against conventional hydroalcoholic ultrasound-assisted extractions. As shown in Figure 1, the free phenolic fraction obtained via hydromethanolic and hydroethanolic UAE yielded TPC values of 2571.83 ± 5.62 µg GAE/mL and 2156.81 ± 19.54 µg GAE/mL, respectively. Among the NaDESs screened, the ChCl:LA mixture emerged as the most effective system, outperforming conventional solvents in terms of extraction yield; interestingly, the extraction efficiency of ChCl:LA was higher at all water content considered. The maximum free TPC was achieved at 20% *v/v* water (3554.27 ± 55.02 µg GAE/mL), representing a substantial increase compared to the hydromethanolic (+64.8%) and hydroethanolic (+38.2%) references. Similarly, when the water concentration was increased to 30% *v*/*v*, the concentration of free phenols was 2776.10 ± 144.44 µg GAE/mL, while at 40% *v*/*v* water, the value increased to 3495.90 ± 6.88 µg GAE/mL. Additionally, promising results were obtained for the recovery of the free phenols using a 40% *w*/*v* water content with Su:CA and Gly:AcS eutectic mixtures, which yielded free phenols concentration values of 1701.26 ± 30.95 and 1803.40 ± 123.81 µg GAE/mL, respectively. The analysis of extraction profiles across the various eutectic mixtures highlights the critical role of water content. Specifically, increasing the water concentration from 20 to 40% *v*/*v* resulted in an enhanced recovery of the soluble fraction from BGP for all evaluated NaDESs, with the exception of ChCl:LA *v*/*v* water. This phenomenon is consistent with several studies in the literature, which emphasize that the solubilization capacity of natural deep eutectic solvents is strongly influenced by the water content in the extraction mixture, together with system viscosity, solubilization mechanisms, and overall mixture polarity [22,35].

In this study, a water concentration range of 20–40% *v*/*v* was selected, as levels exceeding 50% *v*/*v* tend to weaken the hydrogen bond network and disrupt the intermolecular interactions between the hydrogen bond donor and acceptor pairs within the eutectic mixture. Although the addition of water effectively affects the characteristic viscosity of NaDESs, thereby enhancing mass transfer from the plant matrix to the solvent, an excessive water content would lead to a simple aqueous solution of the individual components. This transition would result in a substantial loss of the physicochemical properties of the eutectic system and a subsequent decline in extraction efficiency [36,37,38,39]. Conversely, a low or negligible water content within the eutectic mixture could promote higher solvent viscosity, which hinders the mass transfer mechanisms of solutes from the matrix to the solvent phase, ultimately leading to a decrease in extraction efficiency. Furthermore, the chemical nature of the eutectic mixture, specifically its constitutive components and their respective ratios, significantly impacts solvent viscosity and, consequently, the efficiency in extracting the target analytes. This evidence is supported by the experimental data obtained in this study, as the lowest extraction efficiency for the free phenolic fraction was consistently observed across all eutectic mixtures at the minimum water content of 20% *w*/*v*. Comparable results were observed in our previous study regarding the NaDES-mediated ultrasonic extraction of polyphenols and iridoids from medicinal plant industry by-products, where a ChCl:LA mixture in a ratio 1:5 mixture with a 30% *w*/*v* water content was identified as the optimal system [23]. To the best of our knowledge, no studies currently available in the literature have explored the use of eutectic mixtures for the recovery of phenolic fractions from BGP; specifically, the NaDES systems investigated in this work have not been previously evaluated for this matrix. The only relevant study, conducted by Ji et al., demonstrated the potential of choline chloride-based eutectic mixtures for the delignification of garlic and onion skins. However, that research focused primarily on lignin removal, utilizing a different set of choline chloride-based NaDESs than those presented here [40].

Regarding total polyphenols recovered through ultrasound-assisted extraction followed by alkaline hydrolysis, no significant variations in TPC were observed between the use of ethanol or methanol as extraction solvents. Specifically, the phenolic fraction isolated using hydromethanolic mixtures yielded 4185.55 µg GAE/mL, while hydroethanolic extractions resulted in 3814.30 µg GAE/mL. Comparing these findings with NaDES-mediated ultrasound-assisted extractions followed by alkaline hydrolysis, it is evident that almost all evaluated eutectic mixtures at 40% *v/v* water content, with the sole exception of ChCl:CA, yielded a higher total polyphenol content than conventional hydroalcoholic extractions. Notably, the Su:CA mixture favoured the isolation of a total polyphenol concentration of 4563.74, with a marked prevalence of the bound phenolic fraction (2873.37 ± 0.01 µg GAE/mL), while Gly:AcS yielded 4652.45 µg GAE/mL, again with a predominant bound phenolic of 2849.06 ± 134.12 µg GAE/mL. As previously mentioned, the eutectic mixture ChCl:LA demonstrated particularly promising results. At a 40% *v*/*v* water content, this system yielded the highest total polyphenol content among all evaluated extraction mixtures, reaching a value of 5216.62 µg GAE/mL. Notably, ChCl:LA was the only eutectic system, along with the hydroalcoholic controls, to exhibit superior efficiency in isolating the free phenolic fraction, which remained consistently higher than the bound fraction across all tested water concentrations. It has been postulated that this phenomenon arises not only from the high intrinsic extraction efficiency of the mixture but also from the specific capacity of ChCl:LA to degrade the lignocellulosic matrix of BGP, which promotes partial release of non-soluble phenolics. Several studies are in line with this hypothesis, specifically reporting that eutectic mixtures based on choline chloride and lactic acid in a 1:5 ratio are able to degrade lignocellulosic agricultural by-products [41], such as corncob [42], oil palm empty fruit bunches [43], and wheat straw [44].

### 3.2. DPPH and FRAP Assay for the Determination of Antioxidant Potential

**Figure 2:**
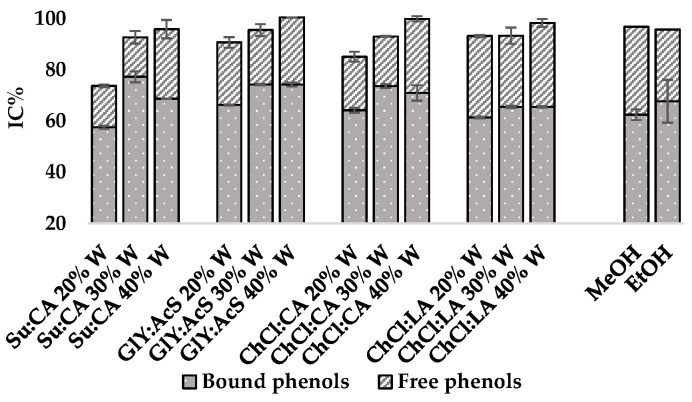
Antioxidant activity in DPPH assay of free phenolic fraction and bound fraction of different NaDESs tested at different water contents (%W).

The soluble phenolic fractions, obtained via ultrasound-assisted extraction using both NaDESs and hydroalcoholic mixtures, as well as the bound fractions recovered through alkaline hydrolysis, were evaluated using two distinct antioxidant assays to determine their radical scavenging potential against different targets; comparison analysis was performed with extracts tested at the same concentration. As illustrated in Figure 2, the contribution of the free phenolic fraction to the overall antioxidant activity was consistently lower than that of the bound phenolic fraction for both NaDESs and conventional hydroalcoholic extracts. Specifically, for the Su:CA extracts, the antioxidant activity of the free phenolic fraction increased (+10.9%) from 16.21% ± 0.51 at 20% *w/v* water content to 27.13% ± 3.60 at 40% *w*/*v*. A similar trend was observed for Gly:AcS and ChCl:CA, which increased (+7.9%) from 20.89% ± 1.94 to 28.86% ± 1.00 over the same water content range. Within this interval, the free phenolic fraction obtained using ChCl:LA reached inhibition capacity values ranging from 31.81% ± 0.43 to 32.83% ± 1.58. These results confirm that ChCl:LA is the most effective extraction medium for isolating the antioxidant components of the BGP matrix. Notably, the antioxidant performance of the free phenolic fraction obtained with ChCl:LA is comparable to that of conventional ultrasound-assisted hydromethanolic extraction (34.35% ± 2.50) and higher than the hydroethanolic analogue (28.01% ± 2.67). Regarding the bound phenolic fraction, the results obtained following alkaline hydrolysis reveal higher contribution to the overall antioxidant capacity compared to the free fraction, as depicted in Figure 2. For all deep eutectic systems, the IC% values associated with the bound fraction remained generally high, exceeding 60%.

Consistent with the trends observed for the free fraction, the antioxidant activity of the bound phenolics extracted via NaDESs showed a general improvement when increasing the water content from 20% to 30% *w*/*v*. As demonstrated, Gly:AcS and ChCl:CA reached values of 74.19% ± 0.29 and 73.63% ± 0.79, respectively, at 30% *w*/*v* hydration. In the case of ChCl:LA, the bound fraction exhibited stable antioxidant values (61.34% ± 0.35 to 65.44% ± 0.30), which, when combined with its superior performance in the free fraction, confirms the excellent efficiency of this lactic acid-based solvent in maximizing the total phenolic recovery from BGP. These findings suggest that the acidic nature of ChCl:LA might promote a preliminary weakening of the lignocellulosic matrix complex during the primary extraction phase, thereby facilitating the subsequent release of bound antioxidants during hydrolysis.

**Figure 3:**
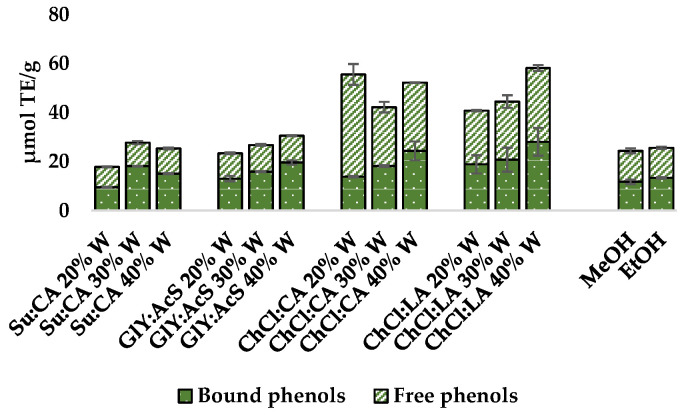
Antioxidant activity in the FRAP assay of the free phenolic fraction and bound fraction of different NaDESs tested at different water contents (%W).

Among the FRAP assay (Figure 3) evaluated for eutectic systems, the ChCl:LA mixture demonstrated high efficacy in extracting antioxidants with high ferric reducing potential. At the maximum hydration level of 40% *w*/*v*, ChCl:LA yielded a bound phenolic reducing power of 28.08 ± 5.70 μmol TE/g, a value that outperforms (+139.4%) the conventional methanolic benchmark (11.73 ± 0.89 μmol TE/g). This result emphasizes the specific ability of the lactic acid-based NaDESs to access the lignocellulosic matrix when compared to traditional organic solvents. Interestingly, the ChCl:LA system exhibited a stable performance across the investigated water content range. In the free phenolic fraction, ChCl:LA reached a peak value of 30.155 ± 1.25 μmol TE/g at 40% *w*/*v* water content. This synergy between the FP and BP fractions suggests that ChCl:LA does not merely act as a solubilizing agent but functions as a reactive extraction medium. Furthermore, the superiority of ChCl:LA over the hydroethanolic control (13.37 ± 0.33 μmol TE/g for BP and 12.27 ± 0.48 μmol TE/g for FP) underscores its potential as a green and efficient alternative for the valorisation of garlic skin by-products. The robust antioxidant profile obtained with this solvent confirms that the specific hydrogen-bonding network established between choline chloride and lactic acid is uniquely suited for the recovery of bioactive antioxidant compounds.

**Figure 4:**
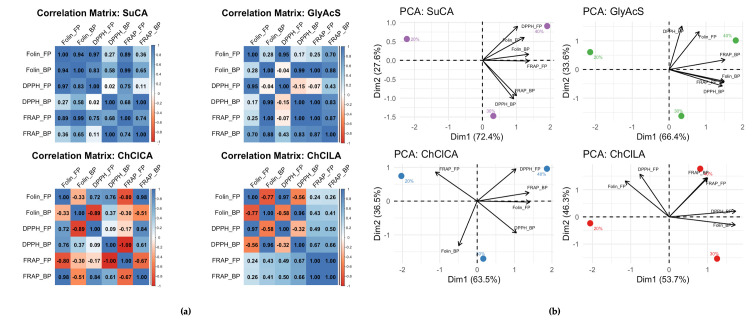
Correlation matrixes (**a**) showing the relationships among Folin–Ciocalteu, DPPH, and FRAP assay responses for different eutectic mixtures and principal component analysis results (**b**).

Correlation analysis and Principal Component Analysis (PCA) were interpreted for each eutectic mixture (Figure 4). Within the free phenolic fraction datasets, the Su:CA and Gly:AcS systems showed generally high positive correlations among the Folin–Ciocalteu, DPPH, and FRAP responses, indicating that free phenolics extracted by these eutectic mixtures drive antioxidant activity. PCA confirmed this trend, with FP samples clustering along a single principal component, reflecting a homogeneous antioxidant profile modulated quantitatively by water content. In contrast, the ChCl-based systems exhibited a distinct multivariate structure. FP in ChCl:LA extracts showed a stronger correlation with the DPPH antioxidant response (r = 0.97) and a weaker correlation with FRAP (r = 0.26). On the other hand, FP values in the ChCl:CA extracts correlated strongly with FRAP (r = 0.98) but weakly with DPPH (r = 0.76). A weaker correlation was observed for bound phenolic fractions where ChCl:CA showed a negative correlation associated with the FP fraction and antioxidant assays (DPPH (r = −0.89) and FRAP (r = −0.51)). In Su:CA, the bound phenolics showed a positive correlation with DPPH (r = 0.58) and FRAP (r = 0.65), while in Gly:AcS eutectic mixtures, the correlation was strong, with r values of 0.99 for DPPH and 0.88 for the FRAP assay. ChCl:LA bound phenolic extracts showed a strong positive correlation with DPPH (r = 0.96) and a positive correlation with the FRAP assay (r = 0.41).

The observed discrepancies in antioxidant response across the different NaDES systems can be attributed to the inherent chemical specificities of the DPPH and FRAP assays. These methods operate through distinct reaction mechanisms and are highly sensitive to the experimental conditions under which they are performed [45]. Specifically, the DPPH assay is typically conducted in an organic medium, which favours the assessment of lipophilic antioxidants or those capable of hydrogen atom transfer [46]. The DPPH assay is also known to be susceptible to interference from other radicals during the determination of antioxidant capacity, which can cause biased results, especially considering the treatment of complex matrices [47]. In contrast, the FRAP assay is carried out under acidic conditions to maintain iron solubility. This low pH environment can induce a partial reduction in the solubility or stability of certain antioxidant species, thereby influencing their electron-donating capacity [28]. It is essential to emphasize that the antioxidant profile of black garlic is characterized by a complex array of non-phenolic metabolites that significantly contribute to the overall radical scavenging and reducing capacity. These compounds include sulphur-containing derivatives such as S-allyl-L-cysteine and S-allylmercaptocysteine, alongside 1,2,3,4-tetrahydro-β-carboline derivatives, pyruvate, melanoidins, and 5-hydroxymethylfurfural [4]. The physicochemical diversity of these non-phenolic species can lead to divergent responses in the DPPH and FRAP assays. Such discrepancies are primarily governed by compounds’ specific solubility in organic versus aqueous media, their structural stability under the varying pH conditions of the tests, and their selective sensitivity toward specific oxidative targets. Consequently, the global antioxidant activity observed in the examined extracts results from a synergistic contribution of both phenolic and non-phenolic fractions, whose individual responses are modulated by the specific experimental conditions of each assay and by the extraction mixture.

### 3.3. Antimicrobial Activity of BGP Extracts

The antimicrobial efficacy of ChCl:LA BGP extract comprising both the free phenolic fraction and the bound phenolic fraction recovered through basic hydrolysis was evaluated against representative bacterial and fungal strains. Due to the nature of the NaDESs, which precludes conventional drying, the minimum inhibitory concentration was expressed as a volume percentage (%*v*/*v*) of the pristine extract serially diluted in water, and as a function of polyphenol content (Table 3). MIC determination was assessed via the CLSI visual macro-dilution method and confirmed by spectrophotometric analysis.

The antimicrobial activity associated with agro-industrial waste, in particular peels generated as residues from different processing stages, is of increasing interest [48]. In the context of this study, the ChCl:LA eutectic BGP extract and ChCl:LA control mixture exhibited the same antibacterial activity against the tested microorganisms. Both the Gram-positive *S. aureus* and the naturally resilient Gram-negative *P. aeruginosa* were completely inhibited at a highly diluted MIC of 0.5% *v*/*v* (corresponding to a 1:200 dilution). The susceptibility of the Gram-negative strain is of particular cosmeceutical interest and is attributed to the ChCl:LA extracting mixture: the lactic acid network within the NaDESs likely acts as an outer membrane permeabilizer, which then exerts antibacterial action.

In this context, recent literature supports the antimicrobial efficacy of NaDESs, particularly those based on organic acids, does not arise from a single mechanism but rather from an interplay of synergistic effects among their constituent components [49]. At the molecular level, the NaDES behaviour characteristic of these systems originates from extensive charge delocalization through hydrogen-bonding networks, which substantially depresses the melting point of the mixture below that of its individual constituents [50]. Charge delocalization has been corroborated by quantum chemical calculations and spectroscopic studies, which demonstrate reduced electron density at hydrogen bond donor sites, confirming the structural reorganization of the eutectic system relative to its pure components. Beyond this physicochemical property, additional mechanisms contributing to the antimicrobial activity of acid-based NaDESs include the solubilization of bacterial membrane constituents, the establishment of a locally acidic microenvironment, elevated osmolality, and the chelation of membrane-associated divalent cations essential for outer membrane integrity, a mechanism of particular relevance in the context of Gram-negative pathogens [51]. In NaDES-based extraction systems, the solvent does not merely act as a passive carrier of phytochemicals but functions as an active co-component with intrinsic antimicrobial properties. This dual functionality, simultaneously acting as a extraction medium that enhances the recovery of bioactive phytochemicals and as an antimicrobially active matrix, represents a distinctive and sustainable advantage over conventional hydroalcoholic extraction systems [52]. Despite the inherent differences in extraction protocol and in the chemical nature of the resulting extracts, the antimicrobial versatility of garlic-derived preparations is well established in the literature. In particular, several studies documented inhibitory potency of garlic extracts against both *P. aeruginosa* and *S. aureus* in association with conventional antibiotics [53] with comparable activity for both microorganisms [54]. Interesting comparative data on the antimicrobial activity of plant-derived extracts reveal that *Punica granatum*, *Syzygium aromaticum*, and *Thymus vulgaris* exhibit similar inhibitory effects against both *P. aeruginosa* and *S. aureus* at a concentration of 10 mg/mL [55]. It should be noted, however, that direct quantitative comparisons between NaDES-based extracts and conventional plant extracts remain inherently challenging, owing to the fundamental differences in extract nature, solvent composition, and the impossibility of expressing NaDES-based extract concentrations in conventional dry weight equivalents, a limitation that must be taken into account when contextualizing the present findings within the broader antimicrobial literature.

Conversely, the antifungal activity against *C. albicans* was significantly lower, requiring a MIC of 50% *v*/*v* (1:2 dilution), which, also in this case, was the same as the NaDES vehicle. This reduced efficacy is consistent with the structural complexity of the yeast cell wall, composed of a thick matrix which inherently provides a more efficient barrier to xenobiotics. Although various studies have documented the antimicrobial properties of FG peels, to the best of our knowledge, the present research is the first to report the combination of BG peels extracted using the ChCl:LA eutectic mixture. Specifically, while conventional FG peel extracts have exhibited inhibitory effects against *S. aureus* [56,57] thereby highlighting their potential application as natural food additives, the synergistic application of extraction media based on choline chloride and lactic acid has previously been reported, as described by [58] specifically against *P. aeruginosa* and *C. albicans* [59].

It must be acknowledged, however, that the scientific literature remains divided on the precise role exerted by the extraction medium itself in determining the antimicrobial activity associated with complex NaDES-based extracts. Disentangling the intrinsic contribution of the solvent system from that of the extracted phytochemicals represents an open methodological challenge that has not yet been resolved consensually in the field. Several studies provide indirect evidence for a solvent-mediated co-contribution to antimicrobial activity. In the context of bioactive extraction from *Humulus lupulus* L., a possible antimicrobial co-action of lactic acid with hop-derived constituents has been postulated, suggesting that the organic acid component of the NaDES matrix may potentiate the activity of co-extracted phytochemicals rather than acting independently [60]. Similarly, Ivanović et al., investigating ChCl:LA-based NaDES extracts of *Achillea millefolium* L., concluded that the enhanced inhibitory activity observed could be associated with the higher content of bioactive compounds recovered by the lactic acid-based system compared to alternative solvents, and also with the intrinsic antimicrobial contribution of the organic acid itself [61]. Conversely, Juneidi et al. reported that NaDESs formulated from choline chloride combined with ethylene glycol, triethylene glycol, or urea exhibited no detectable antibacterial effects, while a mild inhibitory activity was documented exclusively for DES systems based on organic acids, further supporting the hypothesis that the acid moiety, rather than the eutectic system per se, constitutes the primary driver of any solvent-associated antimicrobial activity [62]. Taken together, these findings highlight the complexity of attributing antimicrobial effects in NaDES-based extracts; however, to better understand the specific antimicrobial contributions of the ChCl:LA mixture and the BGP components, further studies should be performed in order to quantitatively assess effects associated with each component. In the specific context of this study and in the experimental conditions in which the antimicrobial assays were performed, the acidic nature of the NaDESs does not allow discrimination of the specific contribution of the antioxidant fraction extracted using the proposed procedure to the biological activity, constituting a limitation of this study. From the comparative analysis of the antimicrobial potential of the ChCl:LA eutectic mixture alone, it emerges that, compared to the BGP extract, the antimicrobial activity is attributable exclusively to the extraction mixture, encouraging more in-depth future evaluation.

### 3.4. HPLC Determination of Phenolic Compounds

The targeted HPLC analysis of the ChCl:LA BGP extracts successfully identified and quantified four key phenolic acids, revealing a phytochemical profile (representative chromatogram reported in Figure S1) that was predominantly characterized by hydroxybenzoic acid derivatives. Interestingly, salicylic acid emerged as the most abundant targeted compound, recorded at a concentration of 0.154 ± 0.003 mg/L, followed by gentisic acid, which was quantified at 0.127 ± 0.001 mg/L (Table 4). Conversely, the targeted hydroxycinnamic acid derivatives were detected at lower concentrations, with chlorogenic acid at 0.034 ± 6.037 × 10^−5^ mg/L and ferulic acid at 0.024 ± 0.008 mg/L. The identification of this specific phenolic distribution provides critical mechanistic insights into the bioactivity of the BGP extracts. Specifically, the high concentration of salicylic and gentisic acids could be indicative of the thermal aging process characteristic of black garlic. Prolonged thermal treatment and the associated Maillard reactions facilitate the breakdown of the lignocellulosic cell wall matrix, promoting the hydrolytic cleavage of esterified bonds and the subsequent release of bound phenolic acids into the extractable fraction [63]. Furthermore, while non-phenolic compounds such as melanoidins and sulphur-containing derivatives contribute to the overall radical scavenging activity, the robust presence of gentisic acid, a hydroquinone derivative with electron-donating capabilities, provides a direct biochemical explanation for the high antioxidant power observed in the extracts.

### 3.5. Photochemiluminescence for the Determination of Antioxidant Activity of Sunscreen Formulations

To comprehensively evaluate the antioxidant potential of the formulated emulsions, a PCL assay was performed, and the results were statistically treated using a one-way ANOVA, followed by Dunnett’s post-hoc test. The observed antioxidant activity (Table 5) was systematically compared against the theoretically expected activity for native ChCl:LA BGP extracts at 5%, 10%, and 15% *w*/*w*. In base formulations (without UV filters), the antioxidant activity at 10% and 15% extract concentrations strictly aligned with the expected theoretical values, confirming a predictable, additive dose-dependent response. A slight, albeit statistically significant, reduction was observed at the lowest concentration of 5% (0.133 vs. 0.180 µmol TE/g, *p* < 0.05). This minor deviation is likely attributable to matrix entrapment effects or minor rheological barriers affecting the extract’s uniform dispersion at lower thresholds, without compromising the overall reliability of the model. Similarly, the incorporation of inorganic UV filters did not alter the antioxidant behaviour of the extracts; all tested concentrations (5%, 10%, and 15%) exhibited experimental values perfectly in agreement with the expected activities (*p* > 0.05), suggesting an inert coexistence between the inorganic particulate and the extract’s bioactive pool. The most noteworthy results emerged from the formulations containing organic UV filters. In these complex matrices, the observed antioxidant activity significantly surpassed the theoretical additive expectations, highlighting a synergistic effect. Specifically, when subtracting the baseline contribution of the vehicle, the synergistic antioxidant boost yielded values 55% higher for the 10% formulation (0.559 µmol TE/g, *p* < 0.01) and higher than 31% for the 15% formulation (0.705 µmol TE/g, *p* < 0.05). This potentiating effect holds substantial cosmeceutical relevance. PCL data reveal the extract’s true role as an advanced secondary defence mechanism. The synergistic interaction observed exclusively with organic filters suggests a beneficial physicochemical interplay between the sunscreen molecules and the NaDES-extracted antioxidant fractions. Organic filters, upon UV absorption, can occasionally generate reactive oxygen species or undergo minor photodegradation. It is highly plausible that the electron-donating compounds within the BGP extract act synergistically to quench these radicals, stabilizing the organic filters, and thus resulting in an amplified overall free-radical scavenging capacity. This positions the NaDES BGP extract as a promising candidate as an active antioxidant booster to mitigate photo-induced oxidative stress in commercial sunscreens.

### 3.6. Photoprotective Activity of Sunscreen Formulations

**Figure 5:**
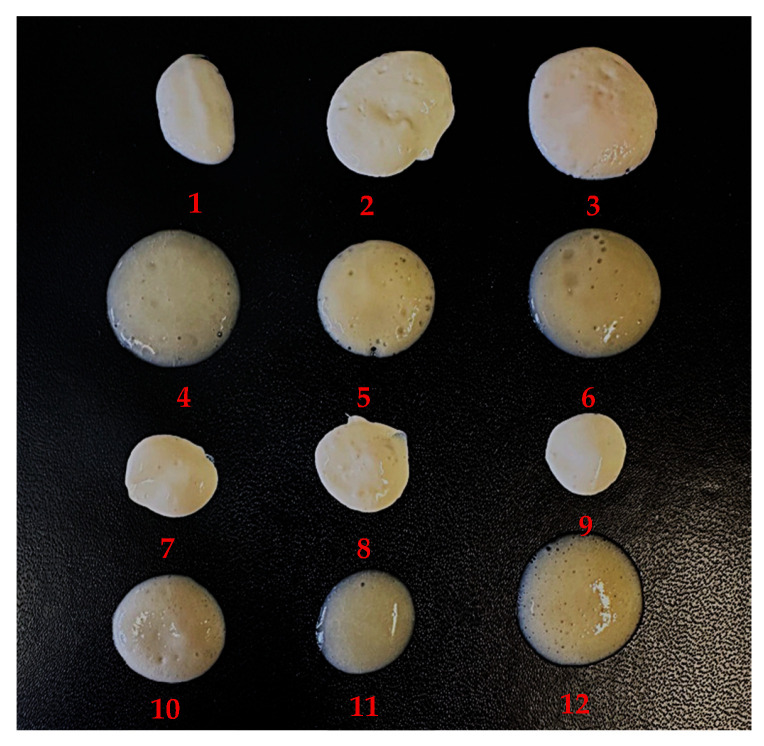
Visualization of sunscreen formulations.

Based on Equations (1) and (2), the SPF and UVA-PF values were determined for all prepared (composition reported in Table 6) formulations (Figure 5). Statistical significance was assessed using a one-way ANOVA, followed by Dunnett’s post-hoc test, comparing each sample against the respective control groups: the blank vehicle (base emulsion without UV filters) and formulations containing UV filters (organic or inorganic) but without ChCl:LA BGP ultrasound-assisted extract. The results demonstrated that formulations enriched solely with BGP extracts at 5%, 10%, and 15% (*w*/*w*) did not yield measurable SPF or UVA-PF values under the experimental conditions.

As expected, these findings indicate that the BGP extract does not exert a direct photoprotective effect or contribute as a primary UV filter to the cosmetic system. Regarding the photoprotective efficacy, formulations containing 10% (*w*/*w*) rutile TiO_2_ (<100 nm) exhibited a baseline SPF of 5.53 ± 0.87 and a UVA-PF of 4.780 ± 1.980, demonstrating a coefficient of variation (CoV) of less than 2.5%. When comparing these baseline metrics to the formulations enriched with the maximum tested concentration (15% *w*/*w*) of the ChCl:LA BGP extract, the resulting SPF and UVA-PF values were recorded at 5.62 ± 0.77 and 5.23 ± 1.50, respectively. Statistical analysis revealed no significant differences between the control and the extract-enriched groups (*p* > 0.05). These findings clearly indicate that the incorporation of the NaDES extract does not physically interfere with the scattering profile of the inorganic UV filter, maintaining the formulation’s primary photoprotective performance unaltered; however, no boosting contribution to the photoprotective activity of titanium dioxide was observed.

Shifting the focus specifically to formulations containing organic UV filters, namely butyl methoxydibenzoylmethane and ethylhexyl salicylate, used in this study, the in vitro assessment revealed no statistically significant enhancement in SPF values upon the addition of the ChCl:LA BGP ultrasound-assisted extract. Compared to the control formulation without the extract (SPF = 6.67 ± 0.35), the incorporation of the extract at 5%, 10%, and 15% resulted in comparable SPF values of 6.520 ± 0.33, 7.42 ± 0.52, and 6.66 ± 0.57, respectively (*p* > 0.05). Notably, however, a distinct functional behaviour was observed in the UVA protection spectrum. While the control formulation yielded a baseline UVA-PF of 12.42 ± 0.87, only the formulation enriched with 10% BGP extract exhibited a statistically significant increase in the UVA-PF, reaching 14.04 ± 1.09 (*p* < 0.001). In contrast, formulations containing 15% *w*/*w* BGP ChCl:LA extract did not produce a statistically significant enhancement of the UVA-PF. This trend in boosting efficacy at the highest extract concentration tested may be attributed to microinstability phenomena arising within the emulsion system, potentially introduced during the pH adjustment step, given the inherently acidic nature of the ChCl:LA eutectic extract and the substantial volume incorporated at 15% *w*/*w*, which may cause physicochemical perturbations within the emulsion microenvironment. Furthermore, the incorporation of an additional hydrophilic component at this concentration level introduces a supplementary aqueous fraction into the final formulation, which may further compromise the uniformity of the thin film applied onto the measurement plate during in vitro SPF determination. Non-uniform film distribution is a well-recognized source of variability in in vitro photoprotective measurements, as local heterogeneities in chromophore concentration directly affect the response and can lead to underestimation or inconsistent quantification of the protective factor. Inhomogeneous chromophore dispersion is known to affect the reproducibility and magnitude of in vitro photoprotective measurements, as the optical response of the thin film applied during SPF determination is highly sensitive to local variations in absorber concentration [31]. This demonstrates a selective UVA-boosting effect, occurring exclusively at the 10% concentration threshold. Although the observed enhancement in UVA protection is statistically significant, the magnitude of this increase remains too modest to justify the primary application of the BGP extract solely as a physical UV-filter booster. Nevertheless, this unexpected functional behaviour paves the way for further in-depth investigations focused on identifying the optimal formulation ratio between specific organic sunscreens, or tailored mixtures, and the exact extract concentration. Such optimization is particularly compelling when considering the consistency between this localized UVA-PF enhancement and the antioxidant synergy demonstrated by the PCL data, suggesting an interdependent mechanism between physical UVA-PF attenuation and biological free-radical scavenging.

## 4. Conclusions

In conclusion, this study successfully employed ultrasound-assisted extraction with various NaDESs to recover polyphenols and antioxidants from black garlic peels. Despite the widespread application of black garlic in the food and nutraceutical sectors, its peels remain an underutilized by-product with immense, yet largely unexplored, valorisation potential. This research highlighted the robust antioxidant profile of the recovered bioactive fractions, establishing the ChCl:LA system with a 40% water content as the optimal green medium for developing cosmeceutical emulsions formulated with either organic or inorganic UV filters. Furthermore, the UAE-derived ChCl:LA BGP extract exhibited significant broad-spectrum antimicrobial activity against key pathogens, including *S. aureus*, *P. aeruginosa*, and *C. albicans*. Further studies will elucidate the effect of extracted bioactive fractions on antimicrobial activity, which in this case is attributable only to the ChCl:LA extraction medium. Interestingly, while the incorporation of the extract did not exert a directly applicable physical boosting effect on the UV-screening of commercial sunscreens, it triggered a synergistic biological antioxidant response when formulated at 10% and 15% in the presence of the evaluated organic filters. To the best of our knowledge, this work represents the first sustainable framework for the valorisation of BGP targeted specifically at cosmetic applications. By successfully bridging green extraction technologies with functional dermatological formulations, it provides a solid foundational platform for future in-depth investigations into the deployment of these innovative NaDES-based extracts in advanced, multi-functional, and sustainable cosmeceuticals.

## Figures and Tables

**Table 1:** NaDESs and relative composition applied to BGP ultrasonic extraction.

Name	Component A	Component B	Abbreviation	Ratio	Water Content [% *w*/*v*]
Sucrose-Citric acid	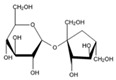	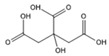	Su:CA	1:2	20 30 40
Glycerol-Sodium acetate	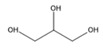	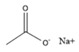	Gly:AcS	3:1	20 30 40
Choline chloride-Citric acid	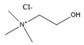	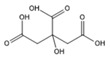	ChCl:CA	2:1	20 30 40
Choline chloride–Lactic acid	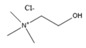	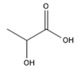	ChCl:LA	1:5	20 30 40

**Table 2:** Ingredients for formulation of sunscreen emulsions.

Component	INCI Name	% *w*/*w*
**Phase A**		
Water	Water	to 100
BGP ChCl:LA extract	-	5 to 15
Dermofeel PA 3	Sodium phytate (and) aqua (and) alcohol	0.1
Glycerin	Glycerin	3
Xanthan gum	Xanthan gum	0.5
**Phase B**		
Montanov 82	Cetearyl alcohol (and) coco-glucoside	3
Cetyl stearyl alcohol	Cetyl stearyl alcohol	2
Myritol 331	Cocoglycerides	4
Cetiol B	Dibutyl adipate	5
Cetiol CC	Dicaprylyl carbonate	8
**Phase C**		
Euxyl K900	Benzyl alcohol (and) ethylhexylglycerin (and) tocopherol	1
UV-filtering agent	Titanium dioxide or butyl methoxydibenzoylmethane and ethylhexyl salicylate	3 to 10
Citric acid or sodium hydroxide	Citric acid/sodium hydroxide	to pH 5.5

**Table 3:** Antimicrobial activity of the ChCl:LA BGP extract and the ChCl:LA control mixture against *S. aureus*, *P. aeruginosa*, and *C. albicans*.

Microorganism	MIC [%*v*/*v*] ^a^	MIC of ChCl:LA [%*v*/*v*] ^a,b^	Extract Dilution Ratio	TPC in Extract [μg GAE/mL]	Susceptibility Profile
*S. aureus*	0.5	0.5	1:200	26.08	Susceptible
*P. aeruginosa*	0.5	0.5	1:200	26.08	Susceptible
*C. albicans*	50	50	1:2	2608.12	Weakly susceptible

^a^ MIC was expressed as %*v*/*v* of pristine extract comprising FP and BP fractions of ChCl:LA BGP ultrasonic extracts. ^b^ MIC of ChCl:LA refers to the antimicrobial activity of the vehicle represented by choline chloride and lactic acid under the same extraction conditions.

**Table 4:** Quantification of phenolic molecules in ChCl:LA BGP extracts via HPLC-DAD.

	Concentration [mg/L]	SD
Chlorogenic acid	0.034	6.037 × 10^−5^
Gentisic acid	0.127	0.001
Ferulic acid	0.024	0.008
Salicylic acid	0.154	0.003

**Table 5:** Comparative analysis of antioxidant potential of sunscreen formulations evaluated via photochemiluminescence. Asterisks refer to *p*-value levels, specifically * *p* < 0.05 and ** *p* < 0.01.

Theoretical Activity	µmol TE/g	Base Emulsion	µmol TE/g	Organic Filter	µmol TE/g	Inorganic Filter	µmol TE/g
5% extract	0.180 ± 0.013	5% extract	0.133 ± 0.004	5% extract	0.302 ± 0.077 *	5% extract	0.1633± 0.006
10% extract	0.359 ± 0.025	10% extract	0.362 ± 0.016	10% extract	0.559 ± 0.005 **	10% extract	0.317 ± 0.021
15% extract	0.539 ± 0.038	15% extract	0.536 ± 0.005	15% extract	0.705 ± 0.020 *	15% extract	0.495 ± 0.034

**Table 6:** Composition of each sunscreen formulation evaluated.

Formulation	TiO_2_ Concentration	Butyl Methoxydibenzoylmethane Concentration	Ethylhexyl Salicylate Concentration	ChCl:LA BGP Extract Concentration	Number
Base emulsion	0	0	0	0	1
10% TiO_2_ emulsion	10	0	0	0	2
3% butyl methoxydibenzoylmethane and 3% ethylhexyl salicylate emulsion	0	3	3	0	3
5% ChCl:LA BGP extract emulsion	0	0	0	5	4
10% ChCl:LA BGP extract emulsion	0	0	0	10	5
15% ChCl:LA BGP extract emulsion	0	0	0	15	6
TiO_2_ + 5% ChCl:LA BGP extract emulsion	10	0	0	5	7
10% TiO_2_ + 10% ChCl:LA BGP extract emulsion	10	0	0	10	8
10% TiO_2_ + 15% ChCl:LA BGP extract emulsion	10	0	0	15	9
3% butyl methoxydibenzoylmethane and 3% ethylhexyl salicylate + 5% ChCl:LA BGP extract emulsion	0	3	3	5	10
3% butyl methoxydibenzoylmethane and 3% ethylhexyl salicylate + 10% ChCl:LA BGP extract emulsion	0	3	3	10	11
3% butyl methoxydibenzoylmethane and 3% ethylhexyl salicylate + 15% ChCl:LA BGP extract emulsion	0	3	3	15	12

## Data Availability

The original contributions presented in this study are included in the article/Supplementary Material. Further inquiries can be directed to the corresponding authors.

## Supplementary Materials

The following supporting information can be downloaded at: https://www.mdpi.com/article/10.3390/antiox15060671/s1. Figure S1: Representative chromatographic profile of diluted ChCl:LA BGB ultrasonic extract acquired in manuscript reported conditions.
