# Supplementary material for: Upcycling Black Garlic Peels into Multifunctional Cosmeceutical Extracts: Antioxidants and UV-Shielding via Antimicrobial Natural Deep Eutectic Solvents
Source: Antioxidants (Basel). 2026 May 27;15(6):671. doi: 10.3390/antiox15060671 (PMC13295795; doi:10.3390/antiox15060671)
Supplement: Supplementary file 1 [file antioxidants-15-00671-s001.zip › antioxidants-4282464-supplementary.pdf]

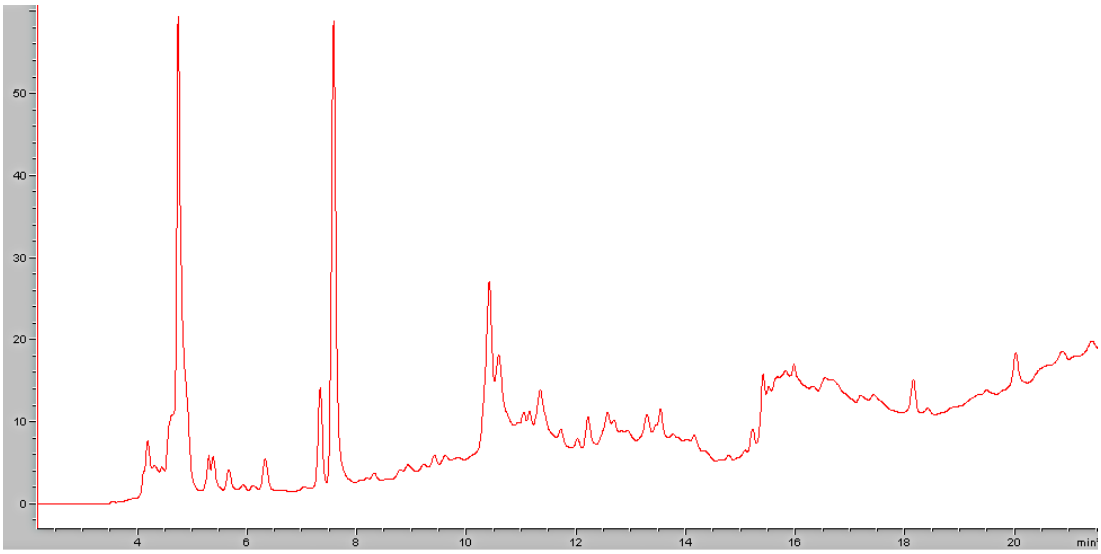

Figure S1. Representative chromatographic profile of diluted ChCl:LA BGB ultrasonic extract acquired in manuscript reported conditions.
